# Spatial variability of bacterial biofilm communities in a wastewater effluent-impacted suburban stream ecosystem

**DOI:** 10.1128/spectrum.04246-23

**Published:** 2024-09-30

**Authors:** Allison M. Veach, Aimee Steinbrecher, Michelle Le

**Affiliations:** 1 Department of Integrative Biology, University of Texas at San Antonio, San Antonio, Texas, USA; Oulun yliopisto, Oulu, Finland

**Keywords:** wastewater, nutrient-diffusing substrata, 16S rRNA, algal biomass, biofilm, lotic

## Abstract

Wastewater discharge is a global threat to freshwater resources. Streams, in particular, are receiving waterbodies that are directly impacted chemically and biologically due to effluent discharge. However, it is largely unknown how wastewater serves as a subsidy or a stressor to aquatic biodiversity, particularly microbiota, over space. Nutrient-diffusing substrata (NDS) were deployed; NDS release nutrients through diffusion into the water column into a wastewater-dependent stream across three reaches. We used N, P, and N + P treatments for the measurement of single nutrient and co-nutrient limitation, and a no-nutrient control. Both algal and total biofilm biomass was measured and the 16S ribosomal RNA genes via targeted amplicon sequencing was used to assess bacterial/archaeal community diversity. Data indicated that total organic matter in biofilms differs spatially with the greatest organic matter (OM) concentrations in the confluence downstream of wastewater inputs. Biofilm OM concentrations were greatest in P and N + P treatments in the confluence site relative to control or N-only treatments. This indicates heterotrophic microbial communities—likely bacteria that dominate stream biofilms—are P-limited in this ecosystem even with upstream wastewater inputs. In conjunction, bacteria/archaeal communities differed the greatest among nutrient treatments versus spatially and had several indicator taxa belonging to *Flavobacterium* spp. in N treatments relative to controls. Collectively with historical water quality data, we conclude that this wastewater-fed stream is primarily N-enriched but potentially P-limited, which results in significant shifts in biofilm bacterial communities and likely their overall biomass in this urban watershed.

**IMPORTANCE:**

Streams in arid and semi-arid biomes are often dependent on their flow from municipal sources, such as wastewater effluent. However, wastewater has been shown to contain high concentrations of nutrients and chemical pollutants that can potentially harm aquatic ecosystems and their biota. Understanding if and the type of microorganisms that respond to pollution sources, specifically effluent from wastewater treatment facilities, in regions where flow is predominantly from treatment facilities, is critical for developing a predictive monitoring approach for eutrophication or other ecological degradation states for freshwaters.

## INTRODUCTION

Freshwater impacted by urbanization is increasing in coverage globally. Approximately 55% of the world population lives in urbanized areas; an additional increase to ~68% residing in urban regions is predicted to occur by 2050 (United Nations, 2018). Indeed, urbanization has resulted in human habitation occurring in 80% of all river basins globally, particularly severe in Europe, North America, Southeast Asia, and Africa (1). Urbanization not only will increase the pressure on freshwater resource use but also likely generate much greater urban and industrial waste, including sewage and wastewater treatment plant (WWTP) effluent. Urban discharges increase both nitrogen and phosphorous nutrient loadings, metals, and novel pollutant mixtures (2, 3).

Wastewater discharged directly into natural waters occurs worldwide (4) and typically results in elevated concentrations of nutrients, novel pollutants, and reduced biodiversity. Although dependent on primary and secondary wastewater treatment processes, wastewater effluent contributes to greater concentrations of several solutes, most notably nitrogen and phosphorous, which lowers water quality and causes ecological degradation of receiving river ecosystems. Wastewater discharge and resulting eutrophication are common in urbanized regions where greater human population numbers will increase sewage loads to treatment plants (3, 5). Advanced treatment processes, such as simultaneous heterotrophic nitrification and aerobic denitrification removal processes, can lower total nutrient loads and pollutant prevalence and increase assimilable substrates for microbial metabolism or biological degradation. For example, implementing activated sludge operations or, similarly, using other biological nutrient removal approaches such as treatment with microalgal biofilms at high retention times may remove large proportions of orthophosphate or nitrate loadings from wastewater (6, 7). However, some advanced treatments such as alternating anaerobic/aerobic conditions to maximize nutrient uptake in wastewater facilities (particularly P-removal) are not cost-effective due to considerable additional labor placed on operations. Even if deployed, treatment facilities may still result in some nutrient enrichment into receiving streams (7). Therefore, wastewater effluent may serve as either a “stressor” or a nutritive “subsidy” for microbial communities in lotic ecosystems receiving wastewater (8). Other work has demonstrated that this effect is largely dependent on the microbial group being studied [algae versus bacteria; specific groups of bacteria (8)] or history of exposure to wastewater and therefore reduced sensitivity to pollutants (9).

Stream biofilms are the primary site of microbial activity and biogeochemical cycling in the stream benthos (10). Within the heterogeneous matrix of microbial cells, the extracellular polysaccharide matrix holds a diverse complex of viruses, bacteria, archaea, algae, fungi, protozoa, among other microbial and smaller-sized macrobial groups. This suite of interacting organisms principally drives whole-ecosystem scale processes through their mediation of nutrients, decomposition, and sometimes, pollutant removal. Waste effluent sources contribute to eutrophication and lead to saturated nutrient uptake by microbial communities (11). This functional shift will also result in compositional shifts for specific microbial groups, particularly bacteria such as Bacteroidetes, notably *Flavobacterium* species, and specific clades of Gammaproteobacteria such as *Pseudomonas* (12). Furthermore, wastewater effluent may also select for greater numbers of dominant taxa and filter or remove rare taxa in streams (12). Urban centers receiving wastewater not only lead to nutrient enrichment but can also lead to declines in microbial diversity in stream sediments (13). Alternatively, wastewater in some contexts may hold high microbial diversity, therefore microbial additions or diversity enrichments to rivers (14). Overall, the impact of wastewater on stream microbial structure and function is an understudied aspect of applied and environmental microbiology.

Addressing nutrient limitation patterns and stochiometric controls for ecosystem processes over space and time through a microbial ecology lens is imperative to understanding the recovery of lotic ecosystems to wastewater discharge. Many studies solely target periphyton growing on artificial substrata to demonstrate such limitation (15–18), although heterotrophic microbes also mediate processes impacting function. Lang et al. (19) demonstrated differing responses of algae and heterotrophic microbial communities to greater N limitation across a nutrient gradient. Likewise, Tank and Dodds (20) also found that algae and fungi in biofilms exhibited different responses to nutrient limitation with fungal P-limitation occurring more frequently than algal P-limitation. The metabolic function of these groups is significantly different, although a proportion of bacteria are photoautotrophic; therefore, differential mechanisms for nutrient limitation are anticipated. Understanding ecosystem-scale responses, such as net energy balance of carbon retention and transport, to anthropogenic change requires the inclusion of both heterotrophic and autotrophic microbial groups’ estimation in future studies. Wastewater effluent may not impact primary production, algal biomass, or algal communities, but instead may impact heterotrophic bacteria, which drive stream respiration, among other diverse functions (8). Therefore, determination of bacterial community composition through gene sequencing or metagenomics is imperative to understand structure-function relationships in response to nutrient limitation in lotic ecosystems.

Intermittent streams and rivers occur globally and are prevalent in semi-arid and arid biomes. In south-central Texas, USA, where intermittency is prevalent among river reaches, several streams depend on wastewater for flow maintenance. However, as discussed previously (21), the presence of wastewater results in excess nutrients, reduced biodiversity, and reduced water quality. Understanding the implications of using wastewater as a source of water to maintain baseflows for lotic ecosystems is essential to determine nutrient mitigation or bioremediation techniques to hopefully lower eutrophication and its impacts on ecosystem health. In this study, a biofilm incubation experiment was performed in a wastewater-dependent tributary of the San Antonio River—Cibolo Creek—to understand how wastewater discharge impacts the biomass and the community composition of microbial communities in the stream benthos spatially downstream of two WWTP point sources.

## MATERIALS AND METHODS

Nutrient-diffusing substrata (NDS) were deployed in three stream reaches in the Upper Cibolo Creek Watershed, north of San Antonio in Boerne, TX, USA. Cibolo Creek lies within the contributing zone of the Edwards Aquifer and is a tributary of the San Antonio River Basin. Within a 2-km area of the Cibolo Creek within Boerne, TX, both a wastewater treatment plant and a more recently built wastewater treatment and recycling center (WWTRC) are present and directly release effluent into Cibolo Creek and its tributaries. Nutrient-diffusing substrata cups were placed immediately downstream of the WWTRC outfall in Menger Creek (referred to as “Menger outfall”), which discharged into Cibolo Creek downstream of the WWTP discharge point and in two downstream reaches below the Menger Creek confluence (referred to as “Cibolo confluence” and “Cibolo downstream” for the most downstream location). The Menger outfall is 260 m upstream of the Cibolo confluence, whereas the Cibolo confluence is 2,035 m upstream of Cibolo downstream (Fig. S1). The Menger outfall and Cibolo downstream were both open canopy sites, whereas the Cibolo confluence was mostly closed canopy (A. Veach, personal observation).

### NDS construction

The NDS were constructed as described in previous work (22, 23) to understand nutrient limitation in this creek spatially. Plastic cups (30 mL polyethylene) were filled with a 2% agar (wt/vol) solution amended with nitrogen (NaNO_3_), phosphorous (KH_2_PO_4_), nitrogen and phosphorous (both NaNO_3_ and KH_2_PO_4_) at 0.5 M, and a no nutrient control with only agar. Each cup had a fritted glass disc placed flush with the solid agar medium to allow for microbial colonization in streambeds. Six replicates of each nutrient treatment were created per site (*N* = 15 each of N, P, N + P, control cups individually, or 60 NDS per site). All NDS cups were secured by cable ties to plastic L-bars, which are plastic pieces in the shape of an “L” when viewed on its side. Two holes were drilled above each NDS placement to allow the cable ties to wrap around the NDS lid. NDS cups were also glued to the bottom of the L bar. The L-bars were then glued to large bricks so that the cups remained upright on the stream bottom during the study. NDS cups were randomly organized onto L-bars so that not all of one-nutrient treatment were placed in one location. NDS cups were placed in a pool at each site on 10 June 2020 and incubated for 26 days. After the incubation period, we collected and transported discs from agar cups on ice in sterile 50-mL centrifuge tubes to the laboratory.

### Historical water quality data collections

Water quality data have been historically collected for one site at the confluence of Cibolo and Menger Creek by the Texas Stream Team’s Program for Surface Water Quality Monitoring. This program collected data at this site monthly, or bimonthly, from June 2019 to June 2020 (1 year previous to this study’s incubation period) for stream discharge (cfs), dissolved oxygen (mg L^−1^), specific conductance (μS cm^−1^), water chemistry [NO_3_^-^, NH_4_^-^, total Kjeldahl N (TKN), total phosphorous (mg L^−1^)], and *Escherichia coli* counts via the most probable number technique (MPN 100 mL^−1^; Table 1; Table S1). Stream discharge was collected using the velocity-area method (24). Point measurements of DO were taken using a YSI 6-Series Optical Membrane sensor and was calibrated using barometric pressure and percent saturation prior to measurement. Specific conductance was measured using a Hydrolab DataSonde 3 and calibrated one point with a potassium chloride (KCl) solution. Water samples were taken for NO_3_^-^, NH_4_^-^, TKN, and TP by immersing acid-washed containers to a depth of 0.3 m. Nitrate, ammonium, and TP samples had one sample (150 mL) collected, whereas TKN had one sample (100 mL) collected in the field. Samples for water chemistry were preserved in a 2:1 H_2_SO_4_ solution and kept at 4°C until analysis with holding times less than 28 days. Nitrate was measured analytically using the EPA Method 353.2 with cadmium reduction. Ammonia was measured using the EPA Method 350.1. TKN was measured using the EPA method 351.2. Total P was measured using the 365.1 EPA Method.

**TABLE 1:** The mean and one standard error of water quality parameters measured 1 year prior to the study period (June 2019–June 2020) in the Cibolo Creek immediately downstream of the Menger Creek confluence, also referred to as our “Cibolo confluence” site

Water quality metric	Sample number	Mean
Discharge (cfs)	9	19.5 ± 11.9
Specific conductance (µS cm^−1^)	10	709.2 ± 58.9
Dissolved oxygen (mg L^−1^)	9	7.0 ± 0.3
Nitrate (mg L^−1^)	9	5.9 ± 1.5
Ammonium (mg L^−1^)	9	0.1 ± 0.001
Kjeldahl nitrogen (mg L^−1^)	6	0.4 ± 0.1
Total phosphorous	9	0.8 ± 0.2
*E. coli* (MPN 100 mL)	8	1,881 ± 896

### Microbial biomass estimates

Both chlorophyll-*a* and biofilm organic matter content from NDS biofilms were measured. Chlorophyll-*a* was measured using the hot ethanol extraction method and determined via spectrophotometry (25). Each disc was individually submerged in ~95% ethanol in an autoclave bag, heated for 5 minutes at 72°C, and then incubated at 4°C in the dark overnight (~16 hours). The following day, bags were removed from the refrigerator and allowed to come to room temperature for 30 minutes while remaining in darkness, prior to analyzing on a Thermo Scientific Biomate-3S UV-Vis Spectrophotometer. Three milliliters of chlorophyll extractant solution and a control ethanol solution were analyzed at 665 and 750 nm.

Biofilm organic matter was determined by measuring ash-free dry mass on disc biofilms. Discs were submerged in 20 mL of sterile water in sterile 50-mL centrifuge tubes and sonicated (40 kHz frequency) for approximately 10 minutes to remove all organic matter from discs. Solutions were then centrifuged at 4,500 rpm/2,300 × *g* for 20 minutes, and supernatant was discarded. The pellet was then aseptically scraped and weighed in aluminum boats, dried at 60°C for 1 week, and then ashed in a muffle furnace (Thermo Scientific Thermolyne Benchtop) at 500°C for 4 hours. The difference between dried and ashed biofilm represents the amount of OM present and is presented as OM g cm^−2^.

### 16S rRNA gene sequencing and bioinformatics

Similar to biofilm OM, biofilms were separated from discs by sonication and then centrifuged at 4,500 rpm for 20 minutes. The pelleted material had genomic DNA extracted subsequently using the PowerBiofilm DNA Isolation Kit (Qiagen, Venlo, the Netherlands) using standard kit procedures except biofilms were bead-beat at 5,500 rpm for 30 seconds three times with a resting stage in between each cycle of 30 seconds using a Precellys-24 Homogenizer. DNA yields were standardized to 3 ng µL^−1^, and an equal molar concentration of DNA (15 ng total) was input in individual PCR reactions for each sample, respectively. PCR assays comprised 313F and 806R primers [0.2 µM final concentration (26)], High-Fidelity Phusion DNA polymerase, dNTPs, and brought up to 25 µL final volume with nuclease-free water. PCR assays were carried out for each sample in triplicate. Thermal cycler parameters included a 5-min denaturation at 94°C followed by 30 cycles of denaturation at 94°C, annealing at 30 seconds at 50°C, and extension for 1 minute at 72°C with final extension for 10 minutes at 72°C. Amplification was confirmed using gel electrophoresis, and a negative control was included in each PCR run to confirm no contamination from reagents or consumables used. Pooled primary PCR assays were then submitted to the University of Texas at San Antonio Integrated Genomics Facility (IGF), where secondary PCRs with Nextera Illumina adapters ligated to Illumina metabarcodes were performed in triplicate. AMPure beads were used to clean PCR amplicons prior to ligating sequence adapters and index barcodes through the Nextera XT indices and NEBNext HiFi Master Mix with eight PCR cycles. The final product was normalized and pooled by concentration and sequenced using the Illumina MiSeq v3 kit through the UTSA IGF.

Paired-end .fastq files demultiplexed via Illumina MiSeq were entered into QIIME2 (27) using the manifest file format. The .fastq files were viewed for quality and then input into the DADA2 algorithm (28). The forward read (R1) was trimmed to 150 bp due to low-quality drop off after 150 bp, and the reverse read (R2) was trimmed to 100 base pairs (bp). Chimera detection was determined using a pooled method among samples and removed as part of the quality-filtering procedure in the DADA2 algorithm. Amplicon sequence variants (ASVs) were created at 99% similarity threshold. We used the SILVA 132 taxonomy database (29) and the Naive Bayes Classifier via the sklearn kit in QIIME2 to determine taxonomic affiliations with each ASV’s representative sequence. This also allowed us to determine contaminants present in the data set (unclassified to Domain, mitochondria under the Alphaproteobacteria class, and chloroplasts under the Cyanobacteria phylum). After quality filtering and contaminant removal, several samples had low sequence yield; therefore, we only retained enough replicates for two of the three study sites to be included in the analysis (Menger outfall, Cibolo confluence), and all samples were subsampled to 2,900 sequences prior to calculating alpha and beta diversity. We also performed a MAFFT alignment and created a phylogeny using FASTTree (30). Alpha diversity estimates include ASV richness, Shannon-Wiener taxonomic diversity, Faiths phylogenic diversity (PD), and Pielou’s Evenness. The rarefied table was then used to calculate additional beta diversity estimates in the R environment. Sequence data are available through the Sequence Read Archive under the BioProject PRJNA1129837.

### Statistical analysis

Two-way analysis of variance (ANOVA) models were used to determine if microbial biomass (chlorophyll-*a* and biofilm OM), bacterial alpha diversity, and bacteria dominant phyla or class differed across NDS nutrient amendments and between sites. Frequency distributions for all response variables were visualized and then Shapiro-Wilks test was performed to test for data normality. Response variables that did not meet normality assumptions were log10-transformed prior to ANOVA models. This included chlorophyll-*a*, biofilm OM, Faiths PD, and the dominant phyla Acidobacteriota, Chloroflexi, Cyanobacteria, Gemmatimonadota, and Myxococcota.

To visualize bacterial/archaeal beta diversity, Bray-Curtis distance matrices were calculated and input into non-metric multidimensional scaling (NMDS) ordination plots. To reduce NMDS stress values, the NMDS had a *k* = 3, although a 2-D plot is provided. A permutational analysis of variance (PERMANOVA) was also calculated using the Bray-Curtis distance matrix of all rarefied ASV counts and site, nutrient amendment, and their interaction was included as independent, predictor variables. These models used 999 permutations by using the *adonis2* function in the vegan package (31). Since nutrient amendment did significantly influence beta diversity, a pairwise comparison for each nutrient amendment group (N, P, N + P, and control) with a false discovery rate *P*-value adjustment was also performed. Finally, to understand indicator taxa of specific amendments among the two retained sites, an indicator species analysis was performed using the indicspecies package in R (32) via the *multipatt* function. Indicator ASVs within one nutrient amendment (N, P, and N + P) versus the control within each site were calculated to determine which species were N or P-limited, or co-limited, and how this differed between sites. An ASV was determined as an indicator at a significance level α ≤ 0.05. The total number of ASVs included in each indicator species analysis was 13,105 across the data set.

## RESULTS

### Water quality

Historical data collected indicated that the Cibolo confluence site had a mean (±1 standard error) discharge of 19.5 ± 11.9 cfs, 7.0 ± 0.3 mg L^-1^ dissolved oxygen, and a specific conductance of 7,092 ± 58.9 µS cm^−1^. Stream NO_3_-N had a mean of 5.9 ± 1.5 mg L^−1^, NH_4_^+^-N was 0.01 ± 0.001 mg L^−1^, and total phosphorous had a mean of 0.8 ± 0.2 mg L^−1^. This site was also confirmed to have *E. coli* levels exceeding recreational waters at a mean of 1,881 ± 896 MPN per 100 mL (Table 1).

### Microbial biomass

**Fig 1:**
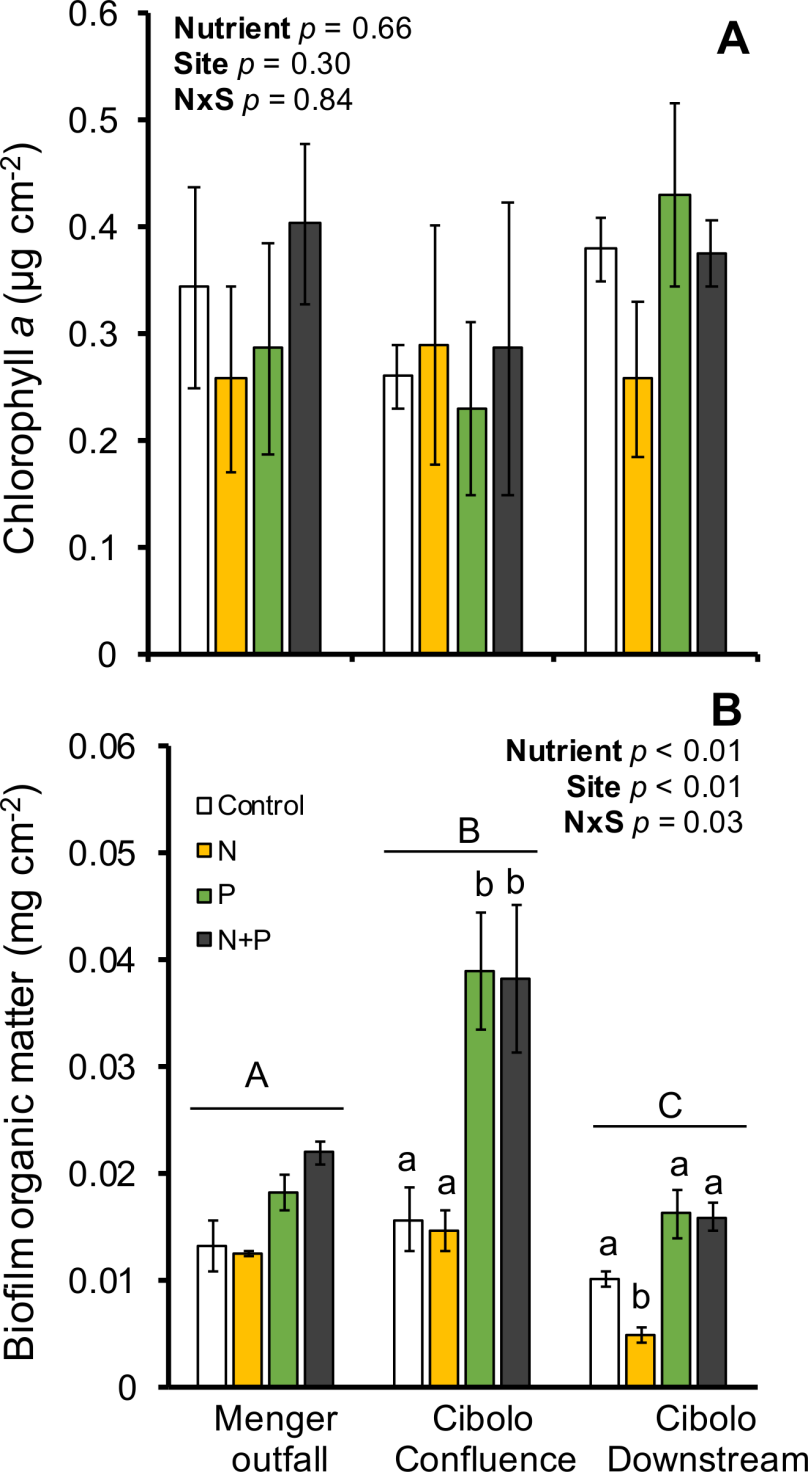
Mean chlorophyll*-a* (A) and biofilm organic matter (B) on NDS discs incubated in three stream reaches impacted by wastewater effluent in Cibolo Creek in Boerne, TX, USA. Bars represent the mean (*N* = 4) ± 1 standard error for each nutrient agar treatment. White bars denote a control with no nutrients, yellow bars denote nitrogen amendments, green bars denote P amendments, and dark gray bars denote N + P amendments. Two-way ANOVA model statistics are provided for both response variables. Biofilm organic matter site pairwise differences are denoted by capital letters, and within site nutrient treatment differences are denoted by lowercase letters.

Chlorophyll-*a* concentrations on substrata did not differ among nutrient treatment (*F*_3,36_ = 0.82, *P* = 0.49), site (*F*_2,36_ = 1.99, *P* = 0.15), or their interaction (*F*_6,36_ = 0.45, *P* = 0.84; Fig. 1A). However, biofilm OM varied by both nutrient (*F*_3,36_ = 30.03, *P* < 0.001), site (*F*_2,26_ = 32.66, *P* < 0.001), and had a significant nutrient by site interaction (*F*_6,36_ = 2.73, *P* = 0.03). Among sites, the Cibolo confluence had the greatest OM relative to other sites and the Menger outfall site also had greater biofilm OM relative to Cibolo downstream (Tukey HSD *P* ≤ 0.001). Biofilm OM only differed among nutrient treatments downstream of the outfall (in the Cibolo Creek sites). Within Cibolo confluence, both P and N + P were significantly greater in OM relative to control treatments (Tukey HSD *P* ≤ 0.003). Within Cibolo downstream, only the N treatment was significantly lower than the control, P, and N + P treatments (*P* ≤ 0.02; Fig. 1B).

### Bacterial alpha and beta diversity

**Fig 2:**
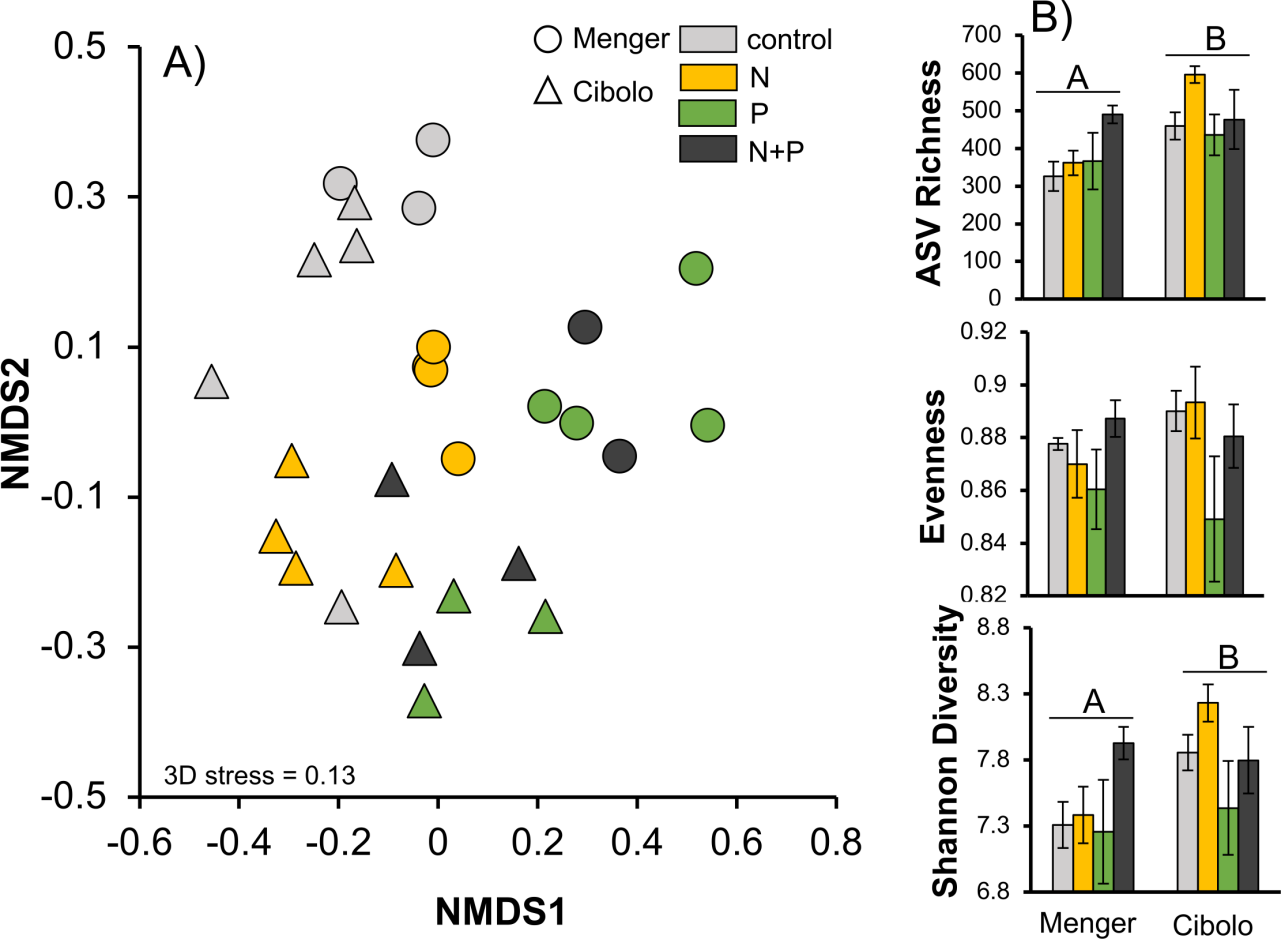
Non-metric multidimensional scaling ordination (A) for bacterial/archaeal communities in stream biofilms amended with N (yellow), P (green), N + P (dark gray), or no nutrient control (light gray) in Menger outfall (circle) and Cibolo Creek confluence sites (triangles). The mean ± 1 standard error ASV richness, Pielou’s evenness, and Shannon diversity (B) across stream biofilms amended with nutrient treatments. Capital letters denote site differences between Menger and Cibolo Creek. The number of replicates varied for nutrient amendments due to subsampling 16S data. Cibolo Creek confluence control had a *N* = 5, nitrogen *N* = 4, N + P and P had *N* = 3, whereas Menger control had *N* = 3, nitrogen and P had *N* = 4, and N + P had *N* = 2.

Site, nutrient amendment, and the interaction between site and nutrient amendment explained a significant amount of variation in bacterial/archaeal community composition (Fig. 2A). The nutrient amendment (*R*^2^ = 0.13, pseudo-*F* = 1.29, *P* = 0.002) and the interaction term (*R*^2^ = 0.13, pseudo-*F* = 1.21, *P* = 0.004) explained more variation in composition than site alone (*R*^2^ = 0.05, pseudo-*F* = 1.32, *P* = 0.01) collectively explaining ~31% of the variation in community composition. Pairwise comparisons with communities exposed to different nutrient amendments indicated that all nutrient amendments were significantly different in composition than no-nutrient controls (PERMANOVA *P* = 0.002; Fig. 2A).

Bacterial ASV richness (*F*_1,19_ = 11.25, *P* = 0.003) and Shannon’s diversity differed among sites (*F*_1,19_ = 6.49, *P* = 0.02) with the confluence downstream having ~28% greater richness (confluence: 494 ± 26, Menger outfall: 374 ± 26) and only ~5% greater diversity (confluence: 7.86 ± 0.12, Menger outfall: 7.41 ± 0.15) than the Menger outfall, respectively (Table 3; Fig. 2B). Nutrient amendment and the interaction between site and nutrient amendment did not differ for richness or Shannon’s diversity (*P* > 0.15). Likewise, Pielou’s evenness and Faith’s phylogenetic diversity did not differ among site or nutrient amendment (*P* > 0.14; Table 3).

### Bacterial taxa distributions

**Fig 3:**
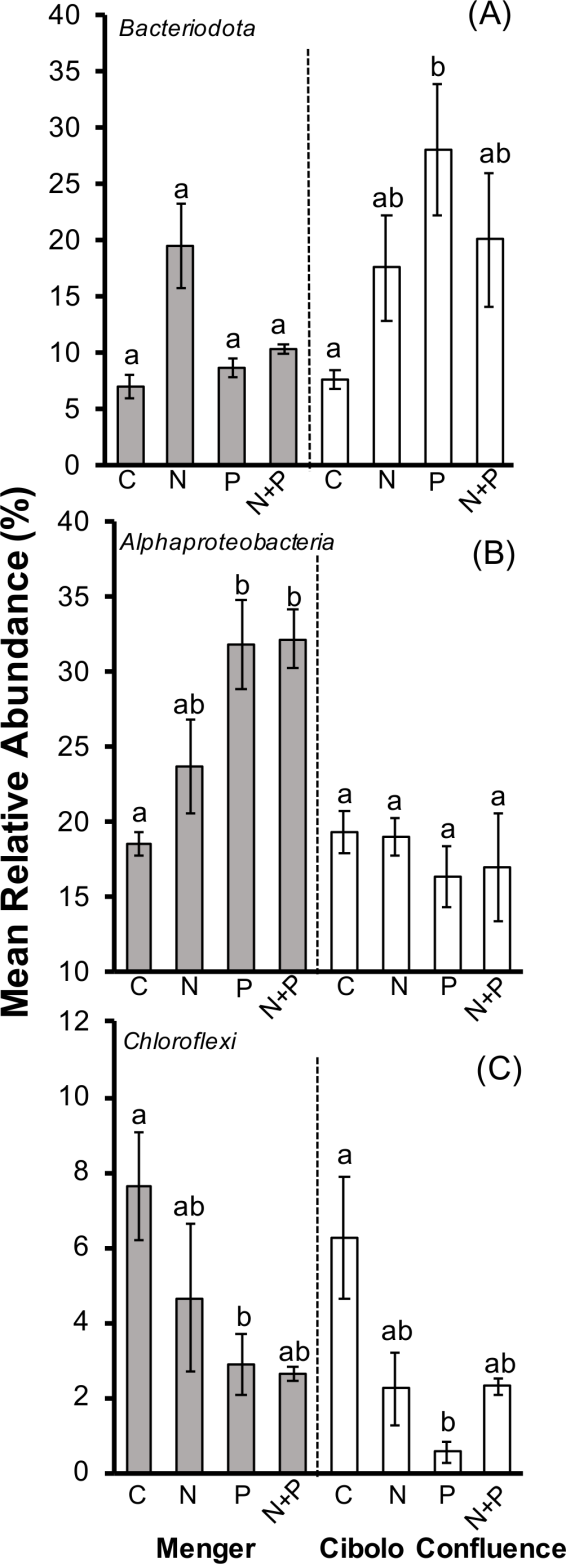
Mean relative abundance of dominant bacterial phyla or class for dominant Proteobacteria, within NDS discs in two stream reaches impacted by wastewater effluent in the Cibolo Creek Watershed in Boerne, TX, USA. Bars represent mean ± 1 standard error for each nutrient agar treatment within each site with Bacteroidota in (A), Alphaproteobacteria in (B), and Chloroflexi in (C). Gray bars denote Menger outfall site and white bars denote the Cibolo Creek confluence site. Lower-case letters denote pairwise statistical differences for a nutrient by site interaction term. Replicates varied for nutrient amendments due to subsampling 16S data. Cibolo Creek confluence control had a *N* = 5, nitrogen *N* = 4, N + P and P had *N* = 3, whereas Menger control had *N* = 3, nitrogen and P had *N* = 4, and N + P had *N* = 2.

There were 42 bacterial or archaeal phyla present among all biofilm samples (Table S2). The dominant phyla (>1% relative abundance) include Proteobacteria (46.4% relative abundance with Alphaproteobacteria comprising 22.7% and Gammaproteobacteria 23.5%), Bacteroidota (14.7%), Actinobacteriota (8.1%), Cyanobacteria (5.6%), Verrucomicrobiota (3.8%), Planctomycetota (3.8%), Chloroflexi (3.5%), Firmicutes (3.2%), Acidobacteriota (2.3%), Gemmatimonadota (1.8%), Myxococcota (1.2%), and Patescibacteria (1.1%; Fig. S2; Table 2). Out of these 13 dominant phyla (or subphyla for Alpha- and Gammaproteobacteria), 6 differed among nutrient treatment, site, or had a significant interaction term, indicating high magnitude structural compositional change spatially and in response to nutrient amendments (Table 2). Both Alphaproteobacteria (*P* = 0.007), Gammaproteobacteria (*P* = 0.01), and Bacteroidota (*P* = 0.03) had a significant nutrient and site interaction (Table 2; Fig. 3). Alphaproteobacteria had greater relative abundance in P (31.7% ± 3.0%) and N + P treatments (32.2% ± 1.4%) relative to control treatments (18.5% ± 0.7%) in the Menger outfall; this site, on average, had greater Alphaproteobacteria relative abundance (26.3% ± 3.6%) compared to the Cibolo confluence (18.2% ± 1.8%; Fig. 3B). Bacteroidota had an opposite trend among sites with relative abundances greatest in the Cibolo confluence (16.8% ± 5.2%) relative to the Menger outfall (11.8% ± 0.9%). Within the Cibolo confluence, the P amendments (28.0% ± 5.1%) had greater relative abundance than the controls (7.7% ± 1.0%; Fig. 3A). Regardless of nutrient amendments, Acidobacteriota (*P* < 0.01), Myxococcota (*P* = 0.51), and Cyanobacteria differed across the two sites. Acidobacteriota and Myxococcota had greater relative abundance in Cibolo confluence (3.8% ± 1.5% and 1.6% ± 0.6%, respectively) relative to Menger outfall (1.1% ± 0.6% and 0.9% ± 0.4%, respectively), whereas Cyanobacteria had greatest abundance in the Menger outfall (9.8% ± 3.0%) relative to Cibolo confluence (3.6% ± 2.7%; Table 2). Regardless of site, Acidobacteriota (*P* = 0.02), Chloroflexi (*P* < 0.01), and Planctomycetota (*P* < 0.01) differed among nutrient amendments (Table 2; Fig. 3). Acidobacteriota had greater abundance in N (2.8% ± 0.7%) relative to P (1.5% ± 1.2%; Tukey HSD *P* = 0.02) and P was weakly lower than control biofilms (3.4% ± 1.6%, Tukey HSD *P* = 0.08). Similarly, Planctomycetota had lower relative abundance in P (2.0% ± 0.7%) and N + P (2.5% ± 0.9%) relative to controls (7.3% ± 2.3%; Table 3). Chloroflexi relative abundance was greater in control (6.8% ± 1.6%) than P amendments (2.0% ± 0.9%; Fig. 3C).

**TABLE 2:** The dominant phyla or class for Proteobacteria (>1% relative abundance) that varied over site (Menger outfall and Cibolo Creek confluence), nutrient treatment, and site x nutrient interaction. Nutrient treatments include N, P, N + P, and a no nutrient control^*a*^

		Site		NDS nutrient		S × N	
Phylum	Relative abundance (%)	*F*-value	*P*-value	*F*-value	*P*-value	*F*-value	*P*-value
**Alphaproteobacteria**	**46.4**	**24.04**	**<0.001**	1.63	0.22	**5.41**	**0.007**
**Gammaproteobacteria**	**22.7**	**6.62**	**0.02**	1.0	0.41	**4.6**	**0.01**
**Bacteroidota**	14.7	3.93	0.06	**5.08**	**0.009**	**3.74**	**0.03**
Actinobacteriota^*b*^	8.1	2.64	0.12	1.46	0.26	2.56	0.08
**Cyanobacteria** ^*b*^	**5.6**	**12.72**	**0.002**	0.92	0.45	1.07	0.39
Verrucomicrobiota	3.8	4.04	0.06	0.29	0.83	0.43	0.73
Planctomycetota^*b*^	**3.8**	0.59	0.45	**6.31**	**0.004**	1.61	0.22
**Chloroflexi** ^*b*^	**3.5**	**4.35**	**0.05**	**7.24**	**0.002**	1.94	0.16
Firmicutes	3.2	2.67	0.12	0.88	0.47	0.43	0.73
**Acidobacteriota^*b*^**	**2.3**	**23.82**	**<0.001**	**4.58**	**0.01**	2.0	0.15
Gemmatimonadota^*b*^	1.8	0.48	0.50	2.01	0.15	0.42	0.74
**Myxococcota^*a*^**	**1.2**	**4.32**	**0.05**	1.91	0.16	1.19	0.34
Patescibacteria	1.1	0.17	0.69	2.24	0.12	0.53	0.67

^*a*^ The model statistics for fixed effects of site, NDS nutrient, and the interaction between site and NDS nutrient used in two-way ANOVA models are included. Bolded phyla indicate those that significantly varied by at least one fixed effect.

^*b*^ phyla log10 transformed prior to analysis.

**TABLE 3:** Alpha diversity metrics that varied over site (Menger outfall and Cibolo Creek confluence), nutrient treatment deployed through NDS, which includes N, P, N + P, and a no nutrient control^*a*^, and site x nutrient interaction.

	Site		NDS nutrient		S × N	
Phylum	*F*-value	*P*-value	*F*-value	*P*-value	*F*-value	*P*-value
**ASV richness**	**11.25**	**0.003**	1.53	0.24	1.94	0.16
Pielou’s evenness	0.98	0.33	2.03	0.14	0.73	0.54
**Shannon’s diversity**	**6.49**	**0.02**	1.39	0.28	1.32	0.30
Faith’s PD	1.69	0.21	1.55	0.23	1.72	0.20

^*a*^ The model statistics for fixed effects of site, NDS nutrient, and the interaction between site and NDS nutrient used in two-way ANOVA models are included. Bolded diversity metrics indicate those that significantly varied by at least one fixed effect. Faith’s phylogenetic diversity (PD) was log10-transformed prior to analysis.

### Indicator ASVs

There were several abundant bacterial ASVs detected in both sites (Table 4). In the Cibolo confluence, nine unique ASVs were identified as indicators, or enriched, in N amendments and were all classified to the *Flavobacterium* genus. Others were detected but were in low abundance and included two in the Alphaproteobacterial *Allorhizobium-Neorhizobium-Pararhizboium-Rhizobium* complex and one indicator ASV in the Gammaproteobacterial *Denitratisoma* genus (Table 4). Two indicator ASVs were found to be P indicators in this site: one also identified as the Alphaproteobacterial *Allorhizobium-Neorhizobium-Pararhizboium-Rhizobium* complex and one in the Actinobacterial *Longivirga* genus. Interestingly, three ASVs were depleted in P amendments relative to a no-nutrient control and included an ASV in Rhizobiales, *Geminocystis* genus in Cyanobacteria, and Xenococcacae Cyanobacterial Family (Table 4).

**TABLE 4:** Indicator species analysis results for bacterial ASVs in control relative to nutrient amendments within a site^*a*^

Site	Treatment	OTU ID	DB classification	BLASTn classification	Identity percentage	Relative abundance
Menger	N enriched	92d52f08631b1cca3d6c9c75d5546610	*Flavobacterium* sp*.^b^*	*Flavobacterium arsenitoxidans*	98.7	0.57
Menger	N enriched	6d81a503435c93d801c371435adaad22	*Flavobacterium* sp.^*b*^	*Flavobacterium arsenitoxidans*	98.5	0.69
Menger	N enriched	f73af83a3348e29aa4ae5e79ae1dbc7d	*Flavobacterium* sp*.^b^*	*Flavobacterium arsenitoxidans*	98.5	0.51
Menger	N enriched	98b7f17d83f8894798c9eb849d37013e	*Flavobacterium* sp*.^b^*	*Flavobacterium arsenitoxidans*	98.7	0.80
Menger	N enriched	03378c88b51b30218150d33b55aef7cf	*Flavobacterium* sp*.^b^*	*Flavobacterium arsenitoxidans*	98.5	0.45
Menger	N enriched	c1e23cc7085a0ced368cf80062f909f7	*Flavobacterium* sp*.^b^*	*Flavobacterium arsenitoxidans*	98.3	0.85
Menger	N enriched	123041f179e329a85bdc9a2e5640f8e1	*Flavobacterium* sp*.^b^*	*Flavobacterium arsenitoxidans*	98.3	0.67
Menger	N enriched	d41b6e786880cb8b5bc2854efa05fd6e	*Flavobacterium* sp*.^b^*	*Flavobacterium arsenitoxidans*	98.5	0.93
Menger	N enriched	887d98f7c933dd6941391bff0d86f32d	*Flavobacterium* sp*.^b^*	*Flavobacterium arsenitoxidans*	98.3	0.46
Menger	P depleted	420aa18467853739c5af6424e28dcaf7	Rhizobiales	uncultured *Hyphomicrobium* sp.	99.3	0.31
Menger	P depleted	81915df0b8408a2522b913e0b6ba5213	*Geminocystis PCC-6308*	*Geminocystis* sp.	99.6	0.19
Menger	P depleted	ecf609127218ef5aaf2101a3a0e1b385	Xenococcaceae	*Chroococcopsis gigantea*	94.6	0.11
Menger	P enriched	ebf041e3ba2183e9656242fdef19e342	*Allorhizobium-Neorhizobium-Pararhizobium-Rhizobium*	*Rhizobium* sp.	99.6	0.11
Menger	P enriched	340b832c44b6e80d6e613aea2641eed0	*Longivirga* sp.	*Longivirga aurantiaca*	99.8	0.20
Down-1	P enriched	ab175f1db53900e720f84d3bdf2ad13c	*Comamonas* sp.	Uncultured *Comamonas* sp.	99.6	0.22

^*a*^ Only indicator ASVs that were ≥0.1% relative abundance were retained. All BLASTn results had an *e*-value of 0.0.

^*b*^ *Flavobacterium* ASVs denoted in Menger for N enrichment were also all indicators for Down-1 for P enrichment. Data not included to minimize repetitiveness.

**Fig 4:**
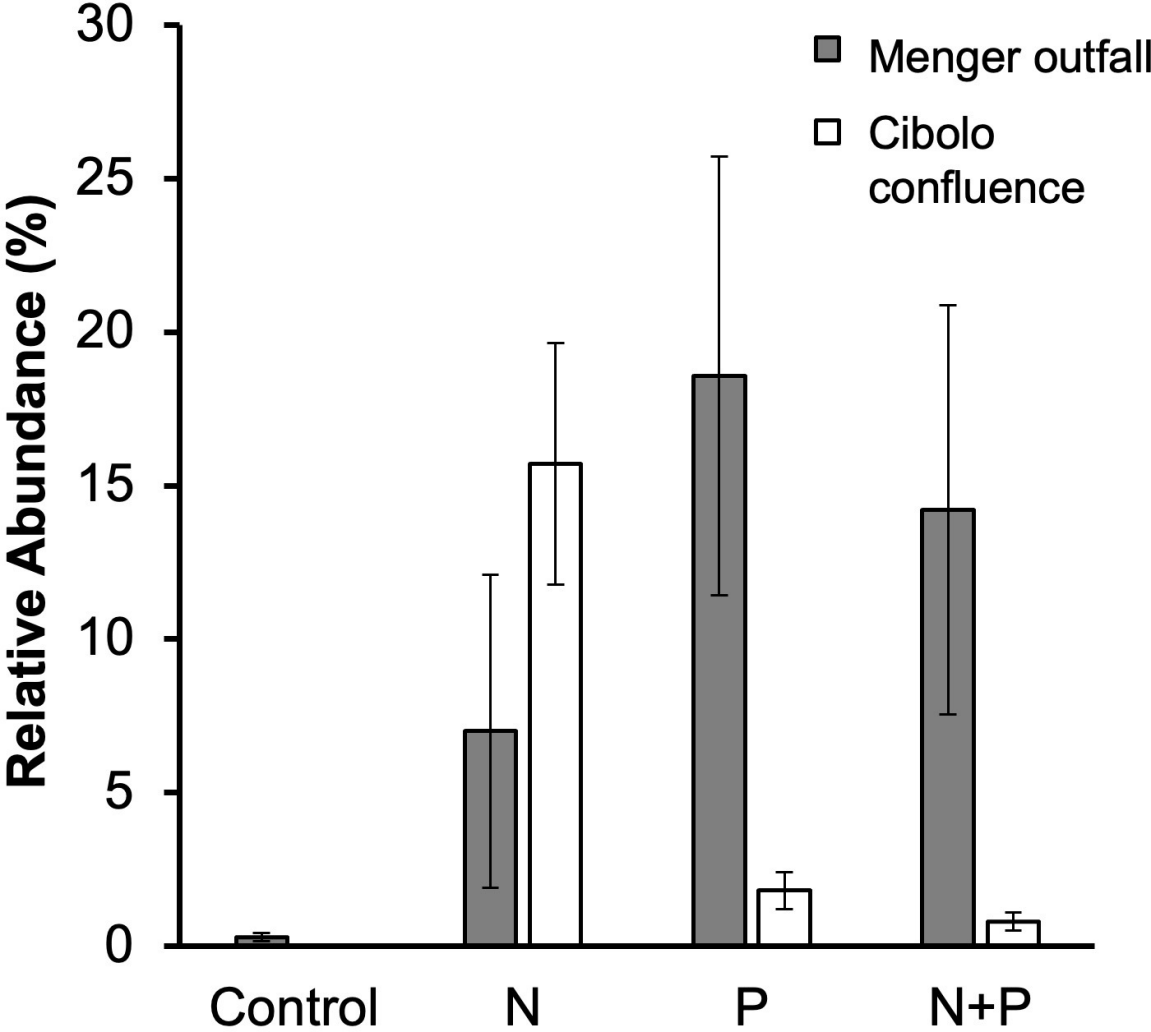
The mean relative abundance of all sequences assigned to the *Flavobacterium* genus in nutrient amendments across sites. Dark gray represents mean abundance in the Menger outfall, and white represents mean abundance in the Cibolo confluence. Bars represent mean ± 1 standard error. Replicates varied for nutrient amendments due to subsampling 16S data. Cibolo Creek confluence control had a *N* = 5, nitrogen *N* = 4, N + P and P had *N* = 3, whereas Menger control had *N* = 3, nitrogen and P had *N* = 4, and N + P had *N* = 2.

In the Menger outfall, all nine *Flavobacterium* ASVs enriched in N amendments in the Cibolo confluence were instead enriched in P (Table 4; Fig. 4) except an additional indicator ASV, the Gammaproteobacterial *Comamonas* species, also was P-enriched. There were three ASVs in low abundance that were N-enriched in this site: one ASV in the Alphaproteobacterial *Devosia* Genus, one in Sphingomonadaceae, and one in the *Allorhizobium-Neorhizobium-Pararhizboium-Rhizobium* complex (Table 4). The relative abundances of all *Flavobacterium* ASVs (446 ASVs assigned to this genus) were enriched with N and P-amended NDS in both the outfall and confluence site (Fig. 4) and are also the most dominant genus across all samples.

## DISCUSSION

Wastewater can serve as a nutrient subsidy or stressor depending on the stream biota and pre-existing water quality states. For example, Aristi et al. (8) indicated that wastewater may act as a subsidy for heterotrophic respiration due to greater OM availability but concomitantly cause a disconnect between photosynthesis and irradiance. This is likely due to wastewater acting as a stressor for algal and photosynthetic bacterial taxa. Likewise, wastewater has been shown to lower bacterial/archaeal diversity in receiving effluent streams while also slowing temperature-mediated decomposition relative to streams without effluent (12). Although this study focuses on microbial biomass and community structure and not on ecosystem functional rates, variable responses are demonstrated. Algal biomass did not vary across sites, yet total biofilm OM was substantially greater in the confluence of the receiving stream and within P treatments, in particular. This suggests that other dominant microbial groups contributing to the OM pool on inert substrata, likely bacteria, may be impacted by nutrient enrichment. Likewise, bacteria increase in biomass due to nutrient limitation unlike algae, and the majority of indicator bacterial ASVs were found in N or P treatments depending on the site. Our results demonstrate that the spatial organization of microbial communities in receiving wastewater effluent streams impacts biofilm bacterial communities and does not impact, at least, algal biomass, although composition may differ spatially.

### Photosynthetic responses to nutrient amendments

This study did not demonstrate differences in algal biomass with nutrient amendments (N or P) and among sites. Although we did not collect water quality samples during the incubation period of this study, historical data indicate that nitrate is high (mean 5.9 mg L^−1^) near the confluence and is likely higher in the outfall that contributes to eutrophication. This is corroborated by our data where N amendments do not elicit greater biomass accrual, either chlorophyll-*a* or OM, on NDS assays (Fig. 1). Other work has demonstrated that nitrogen, not phosphorous, primarily drives algal production, and therefore likely total biomass, in rivers and lakes globally (33). Therefore, it is not surprising that algae did not respond to N or P amendments. Furthermore, light availability may be a stronger driver of algal biomass accrual regardless of nutrient enrichment, particularly when using the NDS experimental approach (34) and in light-limited habitats. Taulbee and Melack (35) demonstrated that algal biomass increases with light availability when nitrogen is present but exhibits opposite trends when only P is added experimentally. Algal biomass responses to nutrient enrichment are likely secondary to other important resources locally; therefore, survey-based results when local site conditions are not similar may be variable in this ecosystem. Menger Creek is open canopy (near the outfall), whereas Cibolo Creek sites where experiments occurred are partially closed canopy. These differences may impact algal biomass data. We posit that neither N nor P may not be the primary driver regulating algal growth or that N and P are supplied in enough concentration that algal communities are not nutrient limited in these impacted streams.

Regardless of the lack of algal responses, other photosynthetic cyanobacterial taxa were found to be depleted in the presence of P in the outfall site, including *Gimnocytis* sp. and *Chrococcopsis gigantea,* which both fix nitrogen and would likely be outcompeted in habitats with abundant available P. Interestingly, other P-depleted indicator taxa include nitrogen fixers, such as *Rhizobium* sp. We did not target other photosynthetic microbial communities that dominate in streams, such as green algae and diatoms, but we expect that differential abundances of these algal groups are likely to mirror the results we see with certain cyanobacterial taxa. In addition, Cyanobacteria did differ in relative abundance across sites with three times greater abundance (~9.8%) in the Menger outfall relative to the confluence. Greater nitrogen concentrations in wastewater bioreactors can lead to selection of cyanobacteria relative to microalgae (36), which may explain the detection of cyanobacterial indicator taxa only in the Menger outfall. Furthermore, the Menger outfall receiving stream is an open canopy, whereas the confluence is more reflective of a natural riparian area where cyanobacteria may not be able to proliferate with lower irradiation levels.

### Bacterial responses to nutrient amendments

Biofilm OM varied spatially and within nutrient amendment treatments in the confluence and downstream of the effluent source in this study. The organic portion of biofilms are comprised of algae, bacteria, some fungi and protozoa, and detritus; algal biomass did not differ spatially or with nutrient treatment; therefore, we posit that the change in OM is likely due in part to changes in bacterial biomass, although this remains speculative. There is evidence that P is relatively low from sewage discharge relative to N (Table 1), which is enriched, and heterotrophic populations responsible for mediating ecosystem function are likely limited in P regardless of wastewater inputs. Bacteria can become P-limited in the presence of abundant N and C concentrations. However, other studies (37) indicate that bacteria are, in general, greater competitors for P relative to algae and therefore are less likely to show differences in growth. However, greater carbon and nitrogen inputs from wastewater effluent will increase bacterial metabolism and therefore increase P-demand (37, 38). This study does not support the idea that bacteria are less likely than algae to exhibit P-limitation based on biofilm OM data. Despite this, this NDS experiment occurred for less than 30 days (22, 39), which is a traditional approach to measure stream nutrient limitation, but this may also have impacts on the time allowed for bacterial and algal colonization. Bacteria colonize inert substrata immediately, whereas it can take algal communities weeks to increase significantly in biomass (40). These results may be partially explained by greater bacterial colonization and growth in a short timeframe or differences in diffusion between P and N treatments using the “plastic-cup NDS” method (39).

Regardless of methodological considerations, results indicate that specific bacterial taxa responded to N and P additions in the effluent outfall site. Several ASVs classified to the genus *Flavobacterium* were “N-enriched” in biofilms grown next to the outfall. Flavobacterium has been detected as an effluent indicator in intermittent, Mediterranean streams (41), temperate urban streams in Japan (42), and urban rivers in Sweden (43). *Flavobacterium* spp. in the phylum Bacteroidetes are a common and abundant heterotrophic bacterial group in freshwater (44, 45) and have been shown to be resistant to antibiotics in wastewater, such as sulfamethoxazole and tetracycline (46). This study demonstrates a high abundance of *Flavobacterium* in the outfall site in particular and suggests that this taxon may serve as a bioindicator in effluent-dominated streams for point sources potentially because it is resistant to common pollutants in wastewater or prefers high levels of N. Accessions found in the BLASTn database indicate that these ASVs are also highly related to *Flavobacterium arsenitoxidans* strains originally discovered as being able to oxidize arsenite (47). Although this does not necessarily indicate that these ASVs are arsenic pollution indicators, it is possible they have unique chemotaxonomic traits allowing for it to degrade wastewater-derived pollutants in the environment.

Bacterial/archaeal alpha diversity varied over site with the confluence harboring the greatest richness and diversity regardless of nutrient amendment. Yet, beta diversity was less affected by spatial location and more so by nutrient amendments. Distributional patterns of bacterial diversity can be influenced by position in the river network (48), and more mechanistically, by changes in dissolved organic matter (DOM) characteristics. DOM has been shown to vary substantially at river confluences, with confluences having greater humic-like DOM (49). We posit that this unique “background” environment regardless of deployed NDS may likely explain why alpha diversity is greater in the confluence in this study. However, we do not replicate these findings across several confluence sites, so this remains speculative. Shifts in bacterial community composition, or beta diversity, were driven by experimental nutrient amendments and the interaction of nutrients with the local reach environment. Similarly, relative abundances of dominant taxa varied by site and nutrient amendment, with certain taxa exhibiting a preference for available P in the outfall (Alphaproteobacteria), whereas an opposite trend was seen in the confluence (Bacteroidota). Acidobacteriota were also N-enriched taxa growing in NDS biofilms. Individual taxa across taxonomic resolutions likely have phylogenetically conserved preferences for a r-strategist or oligotrophic lifestyle. Henson et al. (50) analyzed bacterial communities across the Mississippi River Basin and found that specific groups of OTUs and phyla tended to demonstrate associations with eutrophication status. This is likely also occurring here where, like reference (50), we see that Acidobacteriota prefer high N concentrations and Alphaproteobacteria, if dominant as in the outfall site, may prefer high P concentrations. Additional work must be done in this watershed across several sites, alongside water chemistry measurements, to support this claim more strongly.

### Conclusion

Intermittent rivers in dry climates are dependent on human-supplied sources of water for most of the time, contrary to wetter climates. Treated wastewater effluent is a common type of water source discharged into lotic ecosystems to maintain flow and allow for the survival of freshwater organisms. This study not only uniquely demonstrates disparate responses to nutrient supply for bacteria and algae but also shows that local site conditions or other variables not measured dictate such responses for bacterial communities, regardless of wastewater providing a large portion of stream flow. This study is also limited in that it occurred for a short period (26 days) and only among three reaches of a wastewater effluent-dependent ecosystem. Future research is essential for us to address (i) the mechanisms behind algal nutrient limitation and when nutrients “matter” and (ii) if the results provided here are generally applicable across time and larger spatial extents (among multiple habitats in streams).

## SUPPLEMENTAL MATERIAL

Supplemental material spectrum.04246-23-s0001.docx Fig. S1 and S2; Tables S1 and S2.

ASM does not own the copyrights to Supplemental Material that may be linked to, or accessed through, an article. The authors have granted ASM a non-exclusive, world-wide license to publish the Supplemental Material files. Please contact the corresponding author directly for reuse.
