# Supplementary material for: Spatial variability of bacterial biofilm communities in a wastewater effluent-impacted suburban stream ecosystem
Source: Microbiol Spectr. 2024 Sep 30;12(11):e04246-23. doi: 10.1128/spectrum.04246-23 (PMC11536991; doi:10.1128/spectrum.04246-23)

**Supplementary File**

**
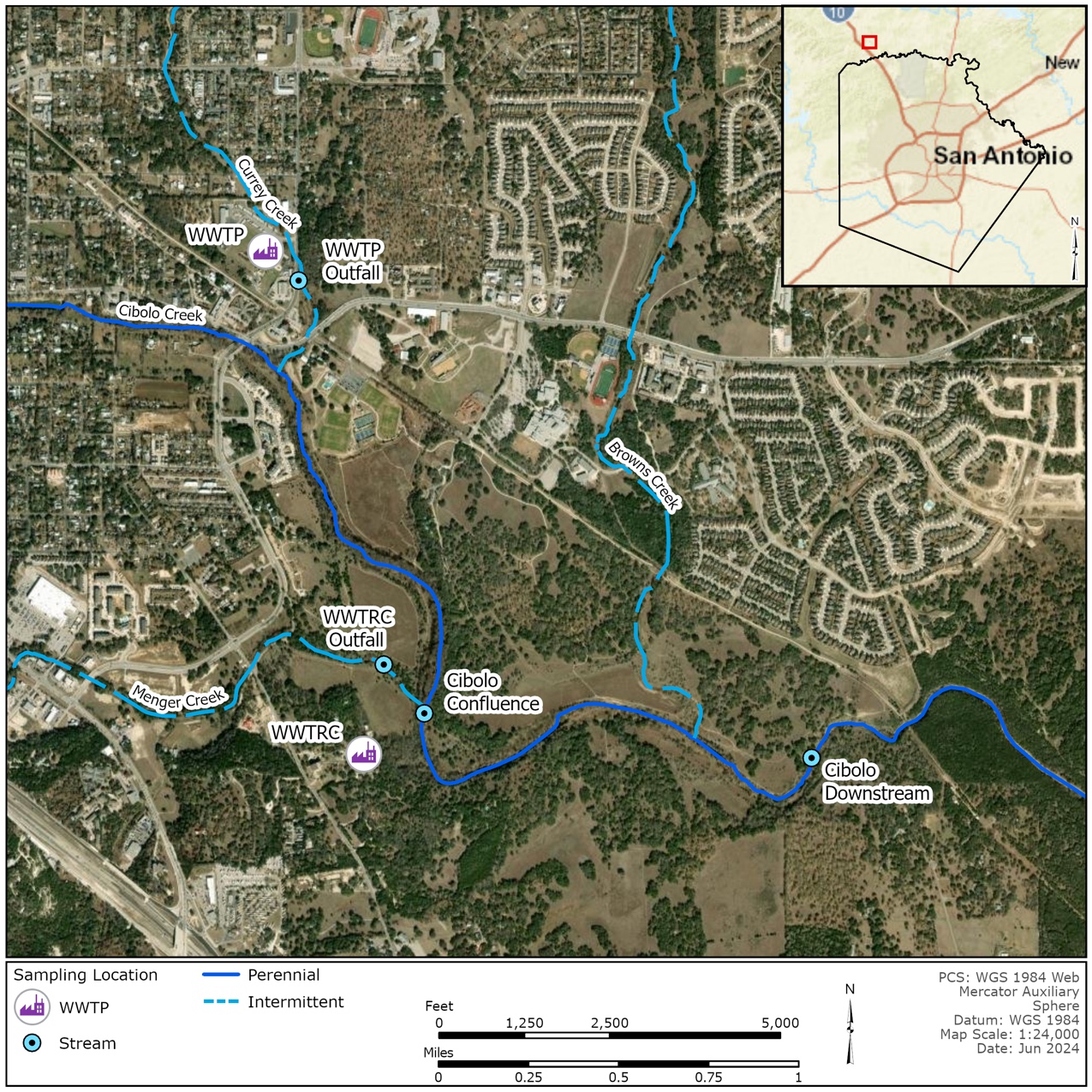
**

**Fig S1.** A map of site locations (WWTRC or Menger Outfall, Cibolo Confluence, and Cibolo Downstream) in Cibolo Creek located in Boerne, TX northwest of San Antonio metropolitan area. The main, perennial channel of Cibolo Creek is denoted in dark blue and intermittent tributaries which only flow either due to wastewater effluent (Menger Creek) or after significant rainfall (Browns Creek) are denoted in a light blue, dashed line.


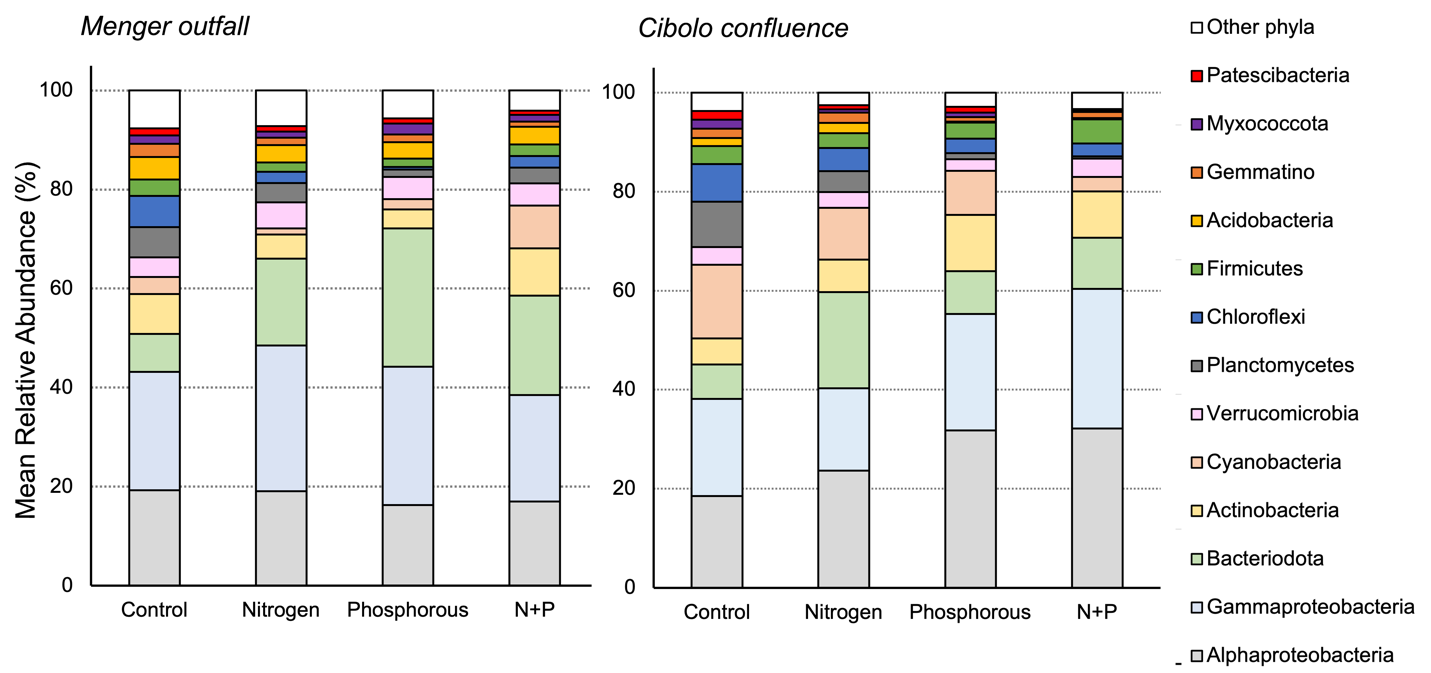


**Fig S2.** The mean relative abundance of dominant (≥ 1%) phyla or class for Proteobacteria across control, nitrogen, phosphorous, and nitrogen + phosphorous (N+P) amended nutrient diffusing substrata (NDS) incubated in the Menger outfall and Cibolo confluence sites near the Cibolo Creek watershed in Boerne, TX, USA. Other phyla include all rare phyla that has a mean abundance of less than 1% in relative abundance in this study.

**Table S1.** Raw data for water chemistry variables in Cibolo Creek 1 year prior to the study. All data provided were collected by the San Antonio River Authority as part of the Texas Stream Team’s Program for Surface Water Quality Monitoring (SWQM). The column, “Value”, refers to the data for each parameter at each time of sampling.

| **Date of Sampling** | **Time of Sampling** | **Parameter** | **Value** |
| --- | --- | --- | --- |
| 5/9/19 | 10:16 | E. COLI, COLILERT, IDEXX METHOD, MPN/100ML | 4100 |
| 6/18/19 | 12:33 | E. COLI, COLILERT, IDEXX METHOD, MPN/100ML | 580 |
| 8/27/19 | 11:43 | E. COLI, COLILERT, IDEXX METHOD, MPN/100ML | 93 |
| 10/3/19 | 9:29 | E. COLI, COLILERT, IDEXX METHOD, MPN/100ML | 160 |
| 12/3/19 | 9:00 | E. COLI, COLILERT, IDEXX METHOD, MPN/100ML | 52 |
| 2/13/20 | 9:30 | E. COLI, COLILERT, IDEXX METHOD, MPN/100ML | 2900 |
| 5/12/20 | 9:40 | E. COLI, COLILERT, IDEXX METHOD, MPN/100ML | 6900 |
| 6/9/20 | 9:58 | E. COLI, COLILERT, IDEXX METHOD, MPN/100ML | 260 |
| 5/9/19 | 10:16 | FLOW STREAM, INSTANTANEOUS (CUBIC FEET PER SEC) | 111 |
| 6/18/19 | 12:33 | FLOW STREAM, INSTANTANEOUS (CUBIC FEET PER SEC) | 33 |
| 8/21/19 | 8:52 | FLOW STREAM, INSTANTANEOUS (CUBIC FEET PER SEC) | 3.3 |
| 8/27/19 | 11:43 | FLOW STREAM, INSTANTANEOUS (CUBIC FEET PER SEC) | 3.3 |
| 10/3/19 | 9:29 | FLOW STREAM, INSTANTANEOUS (CUBIC FEET PER SEC) | 1.9 |
| 12/3/19 | 9:00 | FLOW STREAM, INSTANTANEOUS (CUBIC FEET PER SEC) | 6.2 |
| 2/13/20 | 9:30 | FLOW STREAM, INSTANTANEOUS (CUBIC FEET PER SEC) | 7 |
| 5/12/20 | 9:40 | FLOW STREAM, INSTANTANEOUS (CUBIC FEET PER SEC) | 2.4 |
| 6/9/20 | 9:58 | FLOW STREAM, INSTANTANEOUS (CUBIC FEET PER SEC) | 7.4 |
| 2/13/19 | 1:32 | NITRATE NITROGEN, TOTAL (MG/L AS N) | 1.96 |
| 5/9/19 | 10:16 | NITRATE NITROGEN, TOTAL (MG/L AS N) | 0.665 |
| 6/18/19 | 12:33 | NITRATE NITROGEN, TOTAL (MG/L AS N) | 1.18 |
| 8/27/19 | 11:43 | NITRATE NITROGEN, TOTAL (MG/L AS N) | 8.21 |
| 10/3/19 | 9:29 | NITRATE NITROGEN, TOTAL (MG/L AS N) | 13.2 |
| 12/3/19 | 9:00 | NITRATE NITROGEN, TOTAL (MG/L AS N) | 10.8 |
| 2/13/20 | 9:30 | NITRATE NITROGEN, TOTAL (MG/L AS N) | 3.92 |
| 5/12/20 | 9:40 | NITRATE NITROGEN, TOTAL (MG/L AS N) | 9.06 |
| 6/9/20 | 9:58 | NITRATE NITROGEN, TOTAL (MG/L AS N) | 4.47 |
| 2/13/19 | 1:32 | NITROGEN, AMMONIA, TOTAL (MG/L AS N) | 0.1 |
| 5/9/19 | 10:16 | NITROGEN, AMMONIA, TOTAL (MG/L AS N) | 0.1 |
| 6/18/19 | 12:33 | NITROGEN, AMMONIA, TOTAL (MG/L AS N) | 0.1 |
| 8/27/19 | 11:43 | NITROGEN, AMMONIA, TOTAL (MG/L AS N) | 0.1 |
| 10/3/19 | 9:29 | NITROGEN, AMMONIA, TOTAL (MG/L AS N) | 0.1 |
| 12/3/19 | 9:00 | NITROGEN, AMMONIA, TOTAL (MG/L AS N) | 0.168 |
| 2/13/20 | 9:30 | NITROGEN, AMMONIA, TOTAL (MG/L AS N) | 0.1 |
| 5/12/20 | 9:40 | NITROGEN, AMMONIA, TOTAL (MG/L AS N) | 0.1 |
| 6/9/20 | 9:58 | NITROGEN, AMMONIA, TOTAL (MG/L AS N) | 0.1 |
| 2/13/19 | 1:32 | NITROGEN, KJELDAHL, TOTAL (MG/L AS N) | 0.427 |
| 5/9/19 | 10:16 | NITROGEN, KJELDAHL, TOTAL (MG/L AS N) | 0.64 |
| 6/18/19 | 12:33 | NITROGEN, KJELDAHL, TOTAL (MG/L AS N) | 0.389 |
| 12/3/19 | 9:00 | NITROGEN, KJELDAHL, TOTAL (MG/L AS N) | 0.592 |
| 2/13/20 | 9:30 | NITROGEN, KJELDAHL, TOTAL (MG/L AS N) | 0.425 |
| 6/9/20 | 9:58 | NITROGEN, KJELDAHL, TOTAL (MG/L AS N) | 0.2 |
| 5/9/19 | 10:16 | OXYGEN, DISSOLVED (MG/L) | 7.2 |
| 6/18/19 | 12:33 | OXYGEN, DISSOLVED (MG/L) | 6.9 |
| 8/21/19 | 8:52 | OXYGEN, DISSOLVED (MG/L) | 6.5 |
| 8/27/19 | 11:43 | OXYGEN, DISSOLVED (MG/L) | 6.8 |
| 10/3/19 | 9:29 | OXYGEN, DISSOLVED (MG/L) | 6.5 |
| 12/3/19 | 9:00 | OXYGEN, DISSOLVED (MG/L) | 6.8 |
| 2/13/20 | 9:30 | OXYGEN, DISSOLVED (MG/L) | 9.5 |
| 5/12/20 | 9:40 | OXYGEN, DISSOLVED (MG/L) | 7.1 |
| 6/9/20 | 9:58 | OXYGEN, DISSOLVED (MG/L) | 6.1 |
| 2/13/19 | 1:32 | PHOSPHORUS, TOTAL, WET METHOD (MG/L AS P) | 0.152 |
| 5/9/19 | 10:16 | PHOSPHORUS, TOTAL, WET METHOD (MG/L AS P) | 0.107 |
| 6/18/19 | 12:33 | PHOSPHORUS, TOTAL, WET METHOD (MG/L AS P) | 0.151 |
| 8/27/19 | 11:43 | PHOSPHORUS, TOTAL, WET METHOD (MG/L AS P) | 1.13 |
| 10/3/19 | 9:29 | PHOSPHORUS, TOTAL, WET METHOD (MG/L AS P) | 1.65 |
| 12/3/19 | 9:00 | PHOSPHORUS, TOTAL, WET METHOD (MG/L AS P) | 1.4 |
| 2/13/20 | 9:30 | PHOSPHORUS, TOTAL, WET METHOD (MG/L AS P) | 0.467 |
| 5/12/20 | 9:40 | PHOSPHORUS, TOTAL, WET METHOD (MG/L AS P) | 1.38 |
| 6/9/20 | 9:58 | PHOSPHORUS, TOTAL, WET METHOD (MG/L AS P) | 0.532 |
| 2/13/19 | 1:32 | SPECIFIC CONDUCTANCE,FIELD (US/CM @ 25C) | 592 |
| 5/9/19 | 10:16 | SPECIFIC CONDUCTANCE,FIELD (US/CM @ 25C) | 429 |
| 6/18/19 | 12:33 | SPECIFIC CONDUCTANCE,FIELD (US/CM @ 25C) | 470 |
| 8/21/19 | 8:52 | SPECIFIC CONDUCTANCE,FIELD (US/CM @ 25C) | 840 |
| 8/27/19 | 11:43 | SPECIFIC CONDUCTANCE,FIELD (US/CM @ 25C) | 832 |
| 10/3/19 | 9:29 | SPECIFIC CONDUCTANCE,FIELD (US/CM @ 25C) | 895 |
| 12/3/19 | 9:00 | SPECIFIC CONDUCTANCE,FIELD (US/CM @ 25C) | 936 |
| 2/13/20 | 9:30 | SPECIFIC CONDUCTANCE,FIELD (US/CM @ 25C) | 547 |
| 5/12/20 | 9:40 | SPECIFIC CONDUCTANCE,FIELD (US/CM @ 25C) | 844 |
| 6/9/20 | 9:58 | SPECIFIC CONDUCTANCE,FIELD (US/CM @ 25C) | 707 |

**Table S2.** List of all phyla, both bacteria and archaea, present in this study. *denotes all sequences unclassified to a phylum but classified to Domain Bacteria or Domain Archaea.


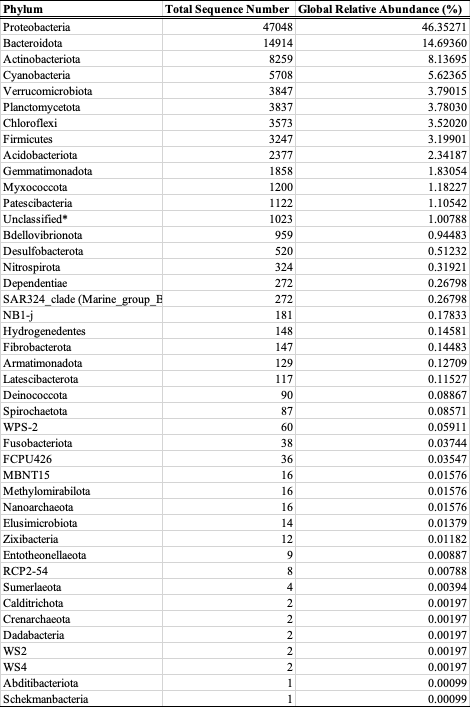

Supplement: Supplemental material — Fig. S1 and S2; Tables S1 and S2. [file spectrum.04246-23-s0001.docx]
